# Supplementary material for: Estimating population size when individuals are asynchronous: A model illustrated with northern elephant seal breeding colonies
Source: PLoS One. 2022 Jan 24;17(1):e0262214. doi: 10.1371/journal.pone.0262214 (PMC8786122; doi:10.1371/journal.pone.0262214)

Estimating the size of a breeding colony of northern elephant seals from a single census: Supporting information

Richard Condit, Sarah G. Allen Daniel P. Costa, Sarah Codde, P. Dawn Goley, Burney J. Le Boeuf, Mark S. Lowry, Patricia Morris

Appendix: Complete Correction factors

Correction factors for northern elephant seals to be applied to daily counts of breeding females. Five locations are included, with three Channel Island colonies combined as one. The total number of females over the entire season is calculated by multiplying a single count on a specified day by the Multiplier listed. Credible intervals (95%) for the estimated total number are derived from the columns Lower and Upper. The correction factors are given separately for the five different sites modeled in the main text: Año Nuevo Island (Table A1), Año Nuevo Mainland (Table A2), Point Reyes (Table A3), Channel Islands (Table A4), and King Range (Table A5).

Table A1. Correction factors for converting daily counts of female northern elephant seals into the total breeding female population at Año Nuevo Island. The total population using the colony in a year is estimated by multiplying a count on any date by the given Multiplier. Lower and upper credible intervals (95%) are derived using the columns Lower and Upper.

Date	Multiplier	Lower	Upper
Jan 11	1.860	1.647	2.184
Jan 12	1.741	1.552	2.022
Jan 13	1.638	1.474	1.878
Jan 14	1.550	1.401	1.761
Jan 15	1.474	1.341	1.661
Jan 16	1.410	1.287	1.581
Jan 17	1.355	1.244	1.508
Jan 18	1.308	1.206	1.445
Jan 19	1.269	1.178	1.393
Jan 20	1.236	1.153	1.348
Jan 21	1.210	1.134	1.310
Jan 22	1.189	1.118	1.282
Jan 23	1.174	1.106	1.259
Jan 24	1.164	1.100	1.246
Jan 25	1.158	1.098	1.242
Jan 26	1.158	1.097	1.242
Jan 27	1.162	1.101	1.249
Jan 28	1.171	1.107	1.265
Jan 29	1.186	1.116	1.289
Jan 30	1.205	1.129	1.316
Jan 31	1.231	1.144	1.351
Feb 1	1.263	1.166	1.393
Feb 2	1.303	1.194	1.445
Feb 3	1.350	1.227	1.504
Feb 4	1.406	1.264	1.575
Feb 5	1.473	1.315	1.660
Feb 6	1.551	1.374	1.755
Feb 7	1.643	1.443	1.868
Feb 8	1.750	1.521	2.002

Table A2. Correction factors for converting daily counts of female northern elephant seals into the total breeding female population at Año Nuevo Mainland. The total population using the colony in a year is estimated by multiplying a count on any date by the given Multiplier. Lower and upper credible intervals (95%) are derived using the columns Lower and Upper.

Date	Multiplier	Lower	Upper
Jan 11	2.379	2.089	3.146
Jan 12	2.177	1.927	2.810
Jan 13	2.006	1.791	2.534
Jan 14	1.860	1.673	2.302
Jan 15	1.736	1.573	2.104
Jan 16	1.629	1.484	1.938
Jan 17	1.537	1.409	1.799
Jan 18	1.459	1.345	1.676
Jan 19	1.392	1.289	1.573
Jan 20	1.335	1.244	1.492
Jan 21	1.286	1.206	1.415
Jan 22	1.245	1.175	1.354
Jan 23	1.211	1.149	1.302
Jan 24	1.183	1.128	1.261
Jan 25	1.161	1.113	1.229
Jan 26	1.144	1.101	1.206
Jan 27	1.132	1.093	1.189
Jan 28	1.125	1.090	1.178
Jan 29	1.123	1.090	1.170
Jan 30	1.125	1.092	1.169
Jan 31	1.132	1.092	1.175
Feb 1	1.145	1.092	1.190
Feb 2	1.163	1.096	1.212
Feb 3	1.188	1.104	1.240
Feb 4	1.219	1.116	1.278
Feb 5	1.258	1.136	1.327
Feb 6	1.306	1.161	1.387
Feb 7	1.363	1.193	1.458
Feb 8	1.433	1.233	1.545

Table A3. Correction factors for converting daily counts of female elephant seals into the total breeding female population at Point Reyes. The total population using the colony in a year is estimated by multiplying a count on any date by the given Multiplier. Lower and upper credible intervals (95%) are derived using the columns Lower and Upper.

Date	Multiplier	Lower	Upper
Jan 11	2.251	1.875	2.682
Jan 12	2.068	1.751	2.428
Jan 13	1.912	1.643	2.216
Jan 14	1.779	1.550	2.042
Jan 15	1.666	1.470	1.893
Jan 16	1.569	1.401	1.774
Jan 17	1.486	1.342	1.673
Jan 18	1.416	1.292	1.589
Jan 19	1.356	1.249	1.512
Jan 20	1.305	1.213	1.447
Jan 21	1.262	1.182	1.392
Jan 22	1.226	1.157	1.346
Jan 23	1.197	1.138	1.306
Jan 24	1.174	1.122	1.272
Jan 25	1.156	1.110	1.245
Jan 26	1.143	1.100	1.225
Jan 27	1.135	1.094	1.208
Jan 28	1.133	1.091	1.199
Jan 29	1.134	1.089	1.198
Jan 30	1.141	1.091	1.207
Jan 31	1.153	1.096	1.225
Feb 1	1.171	1.107	1.248
Feb 2	1.194	1.122	1.281
Feb 3	1.224	1.142	1.322
Feb 4	1.260	1.167	1.372
Feb 5	1.305	1.199	1.432
Feb 6	1.359	1.237	1.505
Feb 7	1.424	1.285	1.594
Feb 8	1.500	1.342	1.701

Table A4. Correction factors for converting daily counts of female northern elephant seals into the total breeding female population at Channel Islands. The total population using the colony in a year is estimated by multiplying a count on any date by the given Multiplier. Lower and upper credible intervals (95%) are derived using the columns Lower and Upper.

Date	Multiplier	Lower	Upper
Jan 11	1.848	1.668	2.000
Jan 12	1.719	1.564	1.866
Jan 13	1.609	1.476	1.752
Jan 14	1.516	1.402	1.655
Jan 15	1.438	1.333	1.567
Jan 16	1.372	1.272	1.491
Jan 17	1.316	1.226	1.425
Jan 18	1.270	1.189	1.369
Jan 19	1.231	1.161	1.322
Jan 20	1.200	1.137	1.281
Jan 21	1.175	1.119	1.248
Jan 22	1.157	1.105	1.220
Jan 23	1.143	1.097	1.198
Jan 24	1.135	1.096	1.182
Jan 25	1.131	1.099	1.171
Jan 26	1.133	1.105	1.167
Jan 27	1.139	1.114	1.169
Jan 28	1.150	1.127	1.174
Jan 29	1.165	1.141	1.188
Jan 30	1.187	1.157	1.210
Jan 31	1.213	1.176	1.241
Feb 1	1.246	1.201	1.282
Feb 2	1.286	1.233	1.331
Feb 3	1.334	1.273	1.388
Feb 4	1.390	1.321	1.457
Feb 5	1.456	1.376	1.539
Feb 6	1.533	1.439	1.634
Feb 7	1.624	1.514	1.745
Feb 8	1.730	1.603	1.875

Table A5. Correction factors for converting daily counts of female northern elephant seals into the total breeding female population at King Range. The total population using the colony in a year is estimated by multiplying a count on any date by the given Multiplier. Lower and upper credible intervals (95%) are derived using the columns Lower and Upper.

Date	Multiplier	Lower	Upper
Jan 11	2.712	2.207	3.376
Jan 12	2.460	2.053	2.992
Jan 13	2.247	1.910	2.673
Jan 14	2.067	1.796	2.433
Jan 15	1.913	1.681	2.223
Jan 16	1.782	1.580	2.048
Jan 17	1.671	1.488	1.907
Jan 18	1.575	1.408	1.779
Jan 19	1.493	1.341	1.679
Jan 20	1.423	1.281	1.592
Jan 21	1.364	1.233	1.516
Jan 22	1.313	1.192	1.455
Jan 23	1.270	1.156	1.402
Jan 24	1.234	1.127	1.360
Jan 25	1.205	1.106	1.323
Jan 26	1.181	1.089	1.290
Jan 27	1.163	1.077	1.268
Jan 28	1.149	1.069	1.247
Jan 29	1.141	1.067	1.242
Jan 30	1.137	1.065	1.235
Jan 31	1.137	1.063	1.241
Feb 1	1.143	1.067	1.258
Feb 2	1.154	1.073	1.270
Feb 3	1.170	1.083	1.290
Feb 4	1.191	1.095	1.323
Feb 5	1.219	1.110	1.366
Feb 6	1.255	1.132	1.412
Feb 7	1.298	1.157	1.472
Feb 8	1.350	1.193	1.541

Appendix 2: Subcolonies at Año Nuevo Mainland

Correction factors and census timing at two subcolonies within the mainland colony at Año Nuevo Mainland, a South Point and a North Point. These are often counted separately. Correction factors, as in the main Appendix, are in Tables A6, A7. Timing of the census at the two mainland subcolonies, plus the Año Nuevo Island colony (which is also graphed in the main text), are shown in Figs A1, A2.

Table A6. Correction factors for converting daily counts of female northern elephant seals into the total breeding female population at Año Nuevo Mainland South. The total population using the colony in a year is estimated by multiplying a count on any date by the given Multiplier. Lower and upper credible intervals (95%) are derived using the columns Lower and Upper.

Date	Multiplier	Lower	Upper
Jan 11	2.419	2.063	2.947
Jan 12	2.212	1.907	2.640
Jan 13	2.036	1.775	2.390
Jan 14	1.886	1.662	2.181
Jan 15	1.758	1.565	2.004
Jan 16	1.648	1.482	1.867
Jan 17	1.554	1.411	1.740
Jan 18	1.474	1.350	1.633
Jan 19	1.405	1.299	1.544
Jan 20	1.346	1.256	1.473
Jan 21	1.296	1.220	1.411
Jan 22	1.254	1.191	1.357
Jan 23	1.218	1.165	1.310
Jan 24	1.189	1.145	1.271
Jan 25	1.166	1.128	1.240
Jan 26	1.147	1.113	1.211
Jan 27	1.134	1.102	1.187
Jan 28	1.126	1.093	1.172
Jan 29	1.122	1.088	1.162
Jan 30	1.124	1.087	1.161
Jan 31	1.130	1.089	1.173
Feb 1	1.141	1.095	1.195
Feb 2	1.158	1.104	1.224
Feb 3	1.181	1.119	1.257
Feb 4	1.210	1.139	1.301
Feb 5	1.247	1.161	1.353
Feb 6	1.293	1.192	1.417
Feb 7	1.348	1.229	1.487
Feb 8	1.414	1.273	1.574

Table A7. Correction factors for converting daily counts of female northern elephant seals into the total breeding female population at Año Nuevo Mainland North. The total population using the colony in a year is estimated by multiplying a count on any date by the given Multiplier. Lower and upper credible intervals (95%) are derived using the columns Lower and Upper.

Date	Multiplier	Lower	Upper
Jan 11	2.458	1.846	3.390
Jan 12	2.222	1.713	2.976
Jan 13	2.025	1.589	2.655
Jan 14	1.861	1.492	2.382
Jan 15	1.724	1.400	2.155
Jan 16	1.609	1.328	1.972
Jan 17	1.512	1.273	1.817
Jan 18	1.431	1.225	1.687
Jan 19	1.363	1.185	1.577
Jan 20	1.306	1.154	1.483
Jan 21	1.258	1.128	1.405
Jan 22	1.219	1.112	1.338
Jan 23	1.188	1.101	1.287
Jan 24	1.162	1.093	1.250
Jan 25	1.143	1.084	1.222
Jan 26	1.130	1.074	1.218
Jan 27	1.121	1.061	1.225
Jan 28	1.117	1.051	1.237
Jan 29	1.118	1.046	1.257
Jan 30	1.123	1.042	1.279
Jan 31	1.133	1.042	1.304
Feb 1	1.149	1.045	1.337
Feb 2	1.169	1.053	1.374
Feb 3	1.196	1.064	1.420
Feb 4	1.229	1.075	1.468
Feb 5	1.270	1.091	1.537
Feb 6	1.320	1.114	1.609
Feb 7	1.379	1.143	1.692
Feb 8	1.449	1.179	1.784

Figure A1. Arrival date at Año Nuevo. Arrival date at three Año Nuevo subcolonies as estimated from the census model (ANI: Año Nuevo Island; ANSP: Año Nuevo South Point); ANNP: Año Nuevo North point). Dashed vertical lines show 95% credible intervals in each year. At the far right, open circles and solid lines show the overall mean (all years combined) at all three subcolonies. See main text Fig 3A.

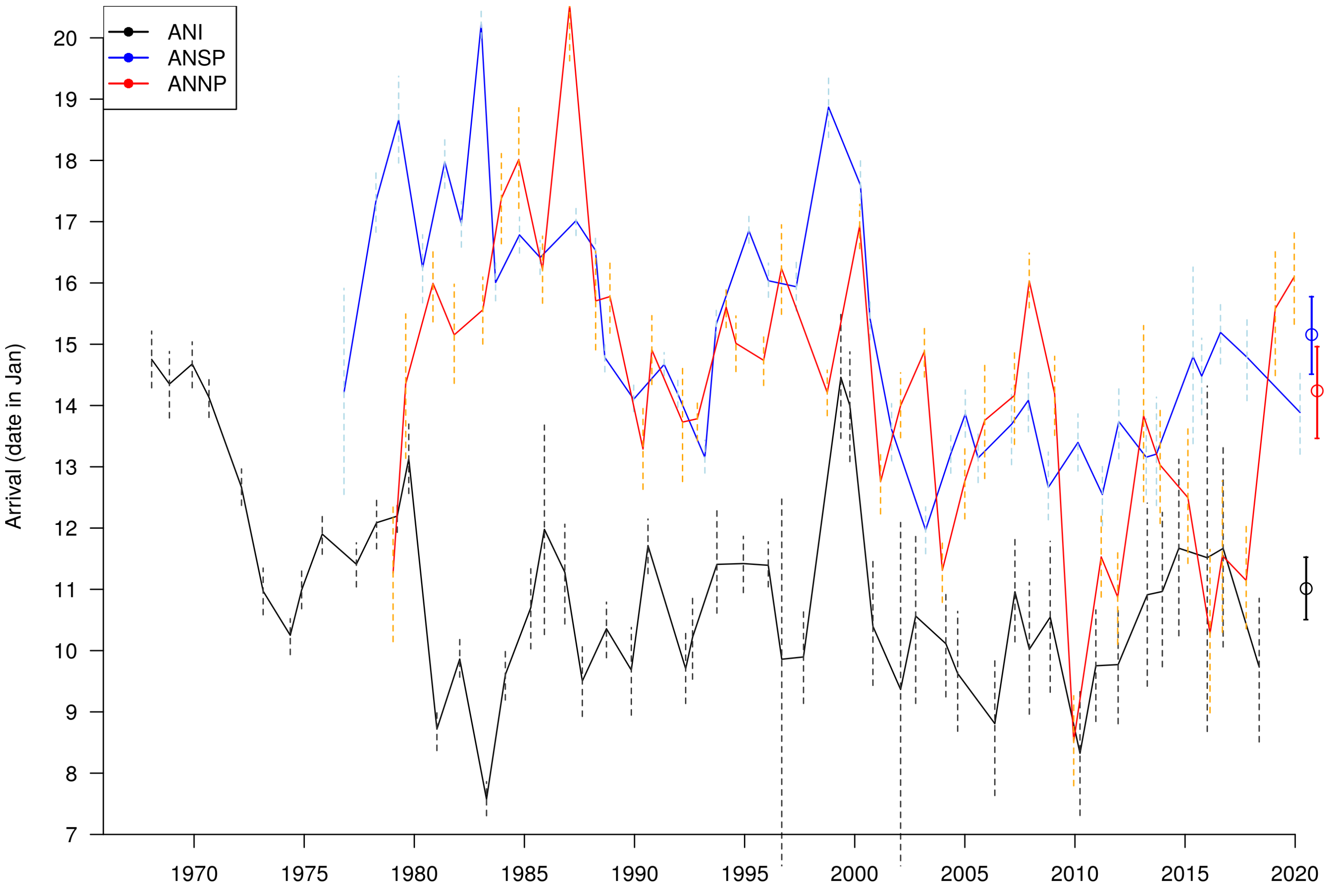

Figure A2. Date of peak census at Año Nuevo. Peak female count at three Año Nuevo subcolonies as estimated from the census model comparing three subcolonies at Año Nuevo (ANI: Año Nuevo Island; ANSP: Año Nuevo South Point); ANNP: Año Nuevo North Point). Dashed vertical lines show 95% credible intervals in each year. At the far right, open circles and solid lines show the overall mean (all years combined) at all three subcolonies. See main text Fig 3B.

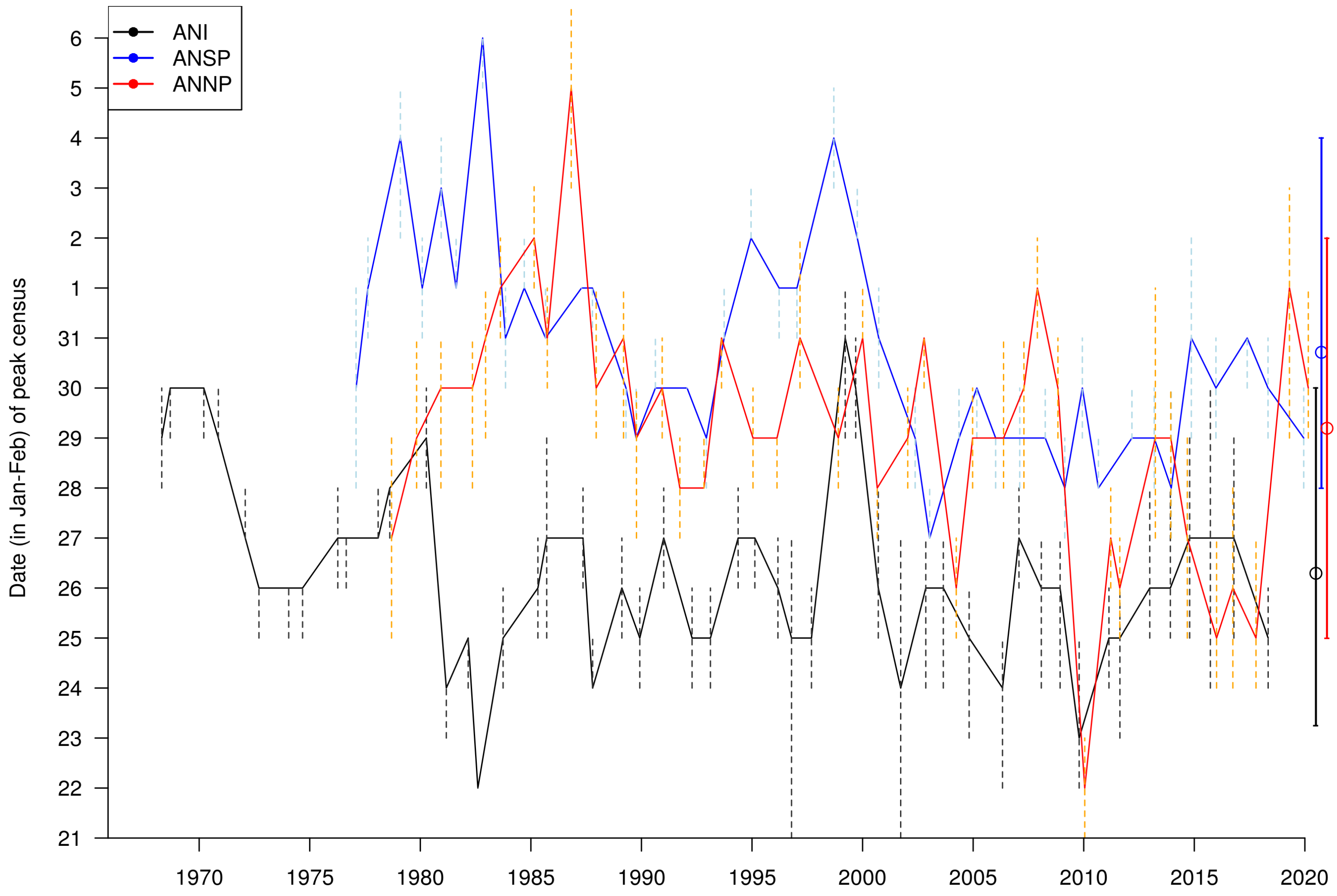

Supplement: S1 Appendix — (PDF) [file pone.0262214.s001.pdf]
