# Supplementary material for: Estimating population size when individuals are asynchronous: A model illustrated with northern elephant seal breeding colonies
Source: PLoS One. 2022 Jan 24;17(1):e0262214. doi: 10.1371/journal.pone.0262214 (PMC8786122; doi:10.1371/journal.pone.0262214)
Supplement: Attachment — Submitted filename: Review (PONE-D-21-03950).pdf [file pone.0262214.s002.pdf]

26/03/2021

Dear *PLoS ONE* Editorial Office,

Review of manuscript PONE-D-21-03950: *Estimating the size of a breeding colony of northern elephant seals from a single census*

The authors have developed a methodology to estimate the annual number of breeding female northern elephant seals at seven study colonies. An impressive dataset has been collected and analysed to accomplish this goal. The premise is simple and the results are of value to the field of population ecology. However, the delivery is very poor. The narrative is not flowing, not enough background information is provided, the language alternates been informal and formal speech, and there many misuses of the English language. Most importantly, the authors have made the paper single-species focussed rather than using their study species as a model organism to develop a methodology that can be applied to other polygynous, capital breeding species. This is not within the scope of *PLoS ONE*.

I found it extremely frustrating that no line numbers were provided in the manuscript. Given that the manuscript could only be downloaded in PDF format, I could not add my own reference system.

Please could the authors address all of my comments and questions in the manuscript as well as in their written response to the reviewer.

<u>Comments</u>

Title:

The objective of this paper was to develop a method of estimating colony size from a single census. I suggest that the authors rephrase the title to make it obvious to the reader that this is a methods paper. For example: “How to estimate breeding colony size of northern elephant seals from a single census”.

Abstract:

Line 1 – I suggest starting the abstract with the problem statement to highlight the importance (the “why”) of this research.

Line 2 – What is difficult? Developing the methodology or censusing elephant seals throughout the breeding season. Please be specific.

Line 3 – Present where? Present at the colony (i.e. land)? Please be specific.

Line 4 – Did you not develop models and opposed to apply pre-existing models? Again, to refer to “those”. Who are “those”? Please be specific.

Line 5 – Replace “yield” with “estimate”. The authors make it seem like the true colony size was calculated as opposed to an estimate with confidence intervals.

Line 5 – “yield total population” what? Population abundance of females? Again, the authors are not specific.

Line 6 – The authors should substitute “California” to the “west coast of North America” for the sake of international readers who do not originate from the USA.

Line 8 – Change “sites” to “colonies” to remain consistent. Simplify “as long as” to “over”.

Line 9 – The optimal correction factor is not a date. Rather it is calculated when the maximum number of females was onshore. Please rephrase and check syntax throughout the manuscript.

Line 10-13 – This section is messy, inconsistent, missing vital information, and can be presented in a simpler manner that is easier to interpret. For example: “The correction factor needed to estimate the **annual number of breeding females** was: (1) 1.15 on 27-28 Jan at Point Reyes, (2)1.17 on 25-26 Jan at A~no Nuevo Island; (3) …”

Please provide dates for the colonies at Channel Island and King Range.

Line 14 – Change to “study colonies”.

Line 14 – “the factor”? The correction factor? A female count? Which females? Adults only? Counts of females in each study colony or all of the colonies combined? Please be specific!

Line 15: Close to optimal? What does this mean? The most accurate? Please be specific.

Line 15-16: Firstly, this should be a separate sentence. Secondly, the authors cannot make this claim without verifying that their method works for unstudied colonies as well. Their findings only apply to their study colonies.

Line 16-18: The entire northern elephant seal population size across demographic age classes or only breeding adult females? Please be specific yet again.

The authors cannot extrapolate their results to all northern elephant seal colonies (see previous comment). The results of this study apply only to the study colonies.

Keywords:

Try to use phrases that are not already in the title of your paper. Include terms that are widely used in the literature.

Introduction:

Line 1-3 – This is not nearly enough information about the life cycle and phenology of northern elephant seals for the general reader to understand the motivation for this study. Not one mention is made about the highly polygynous breeding system of this species which would already give the reader great understanding of the study system.

Line 2 – “raise pups”? Females birth and suckle pups, but there is no paternal care.

Line 3 – Why not breeding males? Please provide details in the manuscript.

Line 3-4 – This has not been written well. Please rephrase and use formal English suited to a scientific journal.

Line 4 – Which models? Population model?

Line 4-5 – The link between the first and second part of this sentence is confusing. Are arrival and departure curves needed to estimate the size of the colony? Perhaps change “and” to “in order to”?

Also, change “on” to “at”. Please check the use of your prepositions throughout the manuscript.

More sentences should be committed here to elaborating the problem statement. The subsequent introduction of the study objective is abrupt and premature. It is here that the authors can also spend more time discussing traditional census methods and how widely these are used for both northern and southern elephant seal censuses. Would the methodology developed by the authors be applicable to other polygynous breeding species? Perhaps mention these here as well so that the paper has broader applicability.

Line 7 – So now there is a “breeding season” which was not mentioned before. For all we know, northern elephant seals come and go on land and breed throughout the year. This is obviously not the case, but the general reader does not have an understanding of northern elephant seal biology prior to this sentence.

Line 8 – Insert total number of *breeding* females. I am sure some sexually mature females skip breeding seasons and remain at sea. Not to mention sexually immature females that do not haul out at all during the breeding season.

Line 8 – “using the colony”? Here the authors are referring to colony as a geographic location (e.g. a beach) but in previous cases colony was used as a collection of breeding females. Please be consistent. Also, what is meant by a colony in this study? What is it comprised of according to the authors’ definition? One or more adult females with pups and a dominant male? Please add your definition to the manuscript.

Line 8-10 – This sentence does not make sense. How are “consistency of census timing and resulting correction factors” related?

Line 10-11 – Please note that it is the methodology developed in this paper which can be applied to other colonies, not the correction factors.

Line 11-12 – Please add “*and is* especially valuable at sites that are difficult to access throughout the breeding season”. I recommend that the authors use the services of an English native speaker to edit the manuscript before resubmitting. I cannot spend time correcting language when I have been asked by the journal editors to review the science of the paper.

Line 12-13 – Why the jump from estimating the annual number of breeding females to annual pup production? The authors are assuming that all pups are successfully weaned each year,

meaning that no pups perish between birth and weaning. Also, the general reader does not know that most females only give birth to a single pup. Please change accordingly.

Line 12-14 – Again, this sentence must apply to the methodology developed by this paper, not the estimated correction factors.

References – the references used in the introduction are outdated. Please cite more recent papers.

Materials and Methods:

Again, no information is provided about northern elephant seal biology, life history, life cycle and phenology – all of which the general reader needs to understand and interpret this paper. Not even the reader can extrapolate the results (methodology) of this study to their study system without this background information.

Field sites:

Line 1 – Insert "*annual* census".

Line 2 – Remove "including all years". Only exceptions need to be mentioned here.

Line 3 – "plus"??? Please use formal language as opposed to informal.

Line 4 – Please add a comma before the first "and".

Line 1-4 – How do these study sites fit into the larger distribution of northern elephant seal breeding colonies? Please specify so that we know what proportion of the total number of breeding colonies were included in this study (and thus the applicability of the results).

Figure 1 – change "analysed" to "studied" in line 2. Similar to my previous comment, the map needs to have a smaller insert so that we know the location of the study colonies in relation the greater distribution of northern elephant seal colonies. Also, this map is poor. It does not have the cardinal points, a scale, key, shading to distinguish land from sea – all the basics which any map should have. In addition, half of the x-axis has been cut off in the manuscript provided. This map adds little value to this paper without the requested information.

Female counts:

Line 1-10 – This section has been written informally and lacks essentially details needed to understand how elephant seals were counted. This is particularly the case for readers with no background in elephant seal biology. Please revise and simplify.

Census model:

Line 1 – Change "through" time to "over" time.

Line 3-4 – What does "modelled rigorously" mean? Please clarify by using standard terminology in the manuscript.

Line 4 – Please add "northern elephant seal *studies*". Please check subject – verb agreement throughout the manuscript.

Change the comma to a semi-colon.

Rather introduce a long list using a colon. I.e. “producing estimates of:”

Employ the services of an editor.

Line 5 – Please add “a(t) and d(t), *respectively*”.

Line 5-9 – There are symbols used in these equations that are not specified (i.e. what do they represent). Overall, the model has been poorly explained. Please try again.

Line 11 – Replace “in” with “by”.

Line 12 – “Here we add one substantial improvement.” Again with the informal speech.

Line 13 – Change to “In previous studies, all years were analysed independently…”.

Line 13 – “estimating a set of parameters for each” what? Please be specific.

Line 15-23 – Far more explanation is needed here for the general reader. Simply referring to Table 1 is not sufficient.

Figure 2

If I assume correctly, Figure 2 is only a subset of the entire dataset with particularly years being extracted for each colony to demonstrate the arrival and departure curve? Why were these particular years chosen? It suggests a bias by the authors. Why not display the graphs of all the study colonies or the same year to eliminate the chances of bias.

Why not just write out the full name of the study colonies above each figure? Why must abbreviations be used? Space does not seem to be limited for these headings.

Please write the first letter of the months (on the x-axis) with a capital letter.

Line 5-8 – This needs to be explained in greater depth in the main manuscript or supplementary material.

Line 7 – “It works”??? Really? Please use formal language.

Table 1

Line 4-5 – What does this mean in simple terms? Either explain it in the methods or supplementary table.

Line 7-8 – This seems more like a statement for the methods section.

Table contents – SD arrival date, Correlation arrival-tenure, Mean tenure on colony (Prior), and CV tenure (Prior) – these are new phrases that are not used or explained in the main manuscript. This feels like an insert from another author who has not paid attention to the narrative of the main text. These abbreviations need to be spelled out in the table header and main text of the methods section.

The correction factor:

Line 4: Change “on” to “at”.

Remove the dot in the equation.

A great job was done in explaining the methods and models of this section. It feels as if a different author wrote this section and inserted it into the main manuscript. The previous sections of the methods need to be modelled on this format of clear, logical, specific, scientific writing. Please use this as your template and seek a “single voice” to obtain a following narrative in the methods section.

Results:

The results section has clearly been written by a different author. This section is written professionally and is without ambiguity. I suggest that the previous sections adopt a similar format to ensure a cohesive manuscript.

Census timing:

Line 3 – Past tense please.

Line 13 – “There were year-to-year fluctuations” in what?

Figure 4 – The abbreviations used here are different to those used in other figures. Please be consistent.

Discussion:

Almost all of the references used in the discussion are outdated. Please cite more recent and relevant papers.

Line 1 – remind the reader of your objectives and overall results before dividing straight into the discussion.

At this point I gave up on counting line number so I will give overarching comments.

More text can be dedicated to placing this study within the context of the current literature than discussing specific details about the study in great length. Has this method been used before or is this the first? What other similar methods have been used for deriving estimates for polygynous, capital breeders? Where can this methodology be applied apart from elephant seals?

There is also a lot of new information about northern elephant seal colonies and biology mentioned for the first time at the end of the discussion. This should never be the case as it leaves the reader confused until the very end. This vital information needs to be introduced earlier in the manuscript.

What other limitations could affect the applicability of this methodology? Climate change and shifting peak haulout dates?

Supplementary table and figure headings:

The authors need to add more details to the headings so that the tables and figures can stand alone and still be confidently interpreted by the reader. These are minor like adding "*northern* elephant seal" but are essential.

Appendix S2:

Line 2 – The authors write South Point and North Point in capital letters implying these are proper nouns. Why then are they introduced as "a" as opposed to "the" if these are established names for these subcolonies?

I may be mistaken but I cannot find any reference to the tables and figures of Appendix S2 in the main manuscript. What is the purpose of this supplementary material? Please make reference to it and explain what was done in the main manuscript.
