# Supplementary material for: Estimating population size when individuals are asynchronous: A model illustrated with northern elephant seal breeding colonies
Source: PLoS One. 2022 Jan 24;17(1):e0262214. doi: 10.1371/journal.pone.0262214 (PMC8786122; doi:10.1371/journal.pone.0262214)
Supplement: Attachment — Submitted filename: Response-to-Reviewers.pdf [file pone.0262214.s003.pdf]

Response to Reviewers
16 November 2021
PONE-D-21-03950

Following Reviewer 1's suggestion that the method be broadened to other species, we substantially rewrote the introduction and discussion to cover asynchronous populations in other species. Most important, we rewrote software to make it available for testing with other datasets. The original source code is published at github, and there is a web portal where data can be uploaded and the program executed, without need for the raw code.

Reviewer 1 also made many suggestions for improving the presentation, and we have followed most. We added background in the Introduction and Methods on basic elephant seal biology, and expanded the statement of the problem, the data available, and the goals of the model. The discussion was rewritten to include a broader context of population estimators in other species, and references were updated. We also removed Appendix 2, which is useful to us but, as noted by Reviewer 1, did not form part of the manuscript. Many other detailed suggestions were incorporated. Here are a few cases where we disagreed:

In some places, Reviewer 1 requested more details about the model, but it was published in full in 2007, and the earlier paper provides details on the parameters and how they are estimated. It would be redundant to repeat them in this paper.

Abbreviations were retained in the figures. In some space was tight, so they were used consistently throughout.

Reviewer 1 suggested we mention polygyny in elephant seals, and they certainly are polygynous. But the problem at hand is estimating the number of breeding females, and we have explained why males are less important (as the Reviewer suggested). But polygyny is not relevant to counts and modeling the population of breeding females.

We did not always agree with Reviewer 1's opinions about English usage. In some cases, the reviewer's preference is not the only correct usage, and we wrote what we think is best.
